# Effects of Cattle Management on Oak Regeneration in Northern Californian Mediterranean Oak Woodlands

**DOI:** 10.1371/journal.pone.0105472

**Published:** 2014-08-15

**Authors:** Aida López-Sánchez, John Schroeder, Sonia Roig, Mar Sobral, Rodolfo Dirzo

**Affiliations:** 1 Departamento de Silvopascicultura, Universidad Politécnica de Madrid, Madrid, Spain; 2 Ecología y Gestión Forestal Sostenible (Research Group), Universidad Politécnica de Madrid, Madrid, Spain; 3 Department of Biology, Stanford University, Stanford, California, United States of America; Institut Pluridisciplinaire Hubert Curien, France

## Abstract

Oak woodlands of Mediterranean ecosystems, a major component of biodiversity hotspots in Europe and North America, have undergone significant land-use change in recent centuries, including an increase in grazing intensity due to the widespread presence of cattle. Simultaneously, a decrease in oak regeneration has been observed, suggesting a link between cattle grazing intensity and limited oak regeneration. In this study we examined the effect of cattle grazing on coast live oak (*Quercus agrifolia* Née) regeneration in San Francisco Bay Area, California. We studied seedling, sapling and adult density of coast live oak as well as vertebrate herbivory at 8 independent sites under two grazing conditions: with cattle and wildlife presence (n = 4) and only with wildlife (n = 4). The specific questions we addressed are: i) to what extent cattle management practices affect oak density, and ii) what is the effect of rangeland management on herbivory and size of young oak plants. In areas with cattle present, we found a 50% reduction in young oak density, and plant size was smaller, suggesting that survival and growth young plants in those areas are significantly limited. In addition, the presence of cattle raised the probability and intensity of herbivory (a 1.5 and 1.8-fold difference, respectively). These results strongly suggest that the presence of cattle significantly reduced the success of young *Q. agrifolia* through elevated herbivory. Given the potential impact of reduced recruitment on adult populations, modifying rangeland management practices to reduce cattle grazing pressure seems to be an important intervention to maintain Mediterranean oak woodlands.

## Introduction

Oak woodland-grasslands have immense ecological and socio-economic importance in Mediterranean regions around the world, supporting both rangeland agroforestry systems and rural populations [1]–[4]. Mediterranean woodland systems are also major biodiversity hotspots [5], [6]. Currently, these systems are characterized by scattered oaks on rolling pastures that support livestock. Historically, Mediterranean woodland systems have been primarily managed for agricultural production with little or no explicitly planned conservation objectives [7]. These woodlands have undergone a variety of land conversion and land-use changes, ranging from low-intensity farmland to extensive irrigation-based agriculture; and from oak woodlands to grasslands or croplands. Such changes threaten Mediterranean biodiversity and ecosystem services [3], [8]. The intensification of land use in Mediterranean agroforestry systems has brought increased soil erosion and reduced forage production [9]–[11]. A sustainable agroforestry system can be combined with appropriately delineated conservation areas to buffer biodiversity loss and maintain ecosystems services [12]. One consequence of current management practices is the apparent lack of oak recruitment occurring in the drier areas of oak ranges [13]–[15]. This trend can lead to long-term declines in natural oak populations [14], [16]. Currently, Mediterranean regions show clear signs of centuries of human habitation and use. Their woodlands support low numbers of young oaks compared to adults, suggesting that populations are not demographically balanced [17].

Californian oak woodlands, or rangelands, are important Mediterranean agroforestry systems that define much of central and northern Californian natural landscapes [18]. They support considerable local biodiversity [18] and are also now threatened on multiple fronts [19]. For decades, the majority of California oak woodlands-grasslands have been used as rangeland for cattle grazing [20]. The causes of poor oak regeneration remain unclear. Among other factors (e.g., acorn diseases or oak death disease; reviewed by [21]), rangeland management practices (especially livestock grazing) have been implicated as primary factors preventing natural oak recruitment [21], [22]. For example, blue oak (*Quercus douglasii* H. & A.) regenerates poorly because the already limited number of established seedlings does not survive into the sapling class [23], [24], and one know cause of blue oak recruitment limitation is livestock grazing, mainly cattle [25]. On the other hand, valley oak (*Quercus lobata* Née) seedlings seem to able to tolerate dry habitats [26] and deer browsing [18] better than coast live oak (*Quercus agrifolia* Née), whereas coast live oak seedlings and saplings usually do not appear in open habitat but can be found under some shrubs, presumably because there they find refuge from wildlife (mainly deer) or livestock grazing [17]. The establishment of first-year-coast live oak seedlings depends on rainfall levels (as they must survive prolonged summer drought), soil conditions, and levels of seed predation by small mammals [18]. It has been suggested that deer and cattle browsing are relatively minor factors of mortality for coast live oak seedlings, but become more important as individuals transition to the sapling stage [18]. Cattle generally cause more shoot damage because they typically chew into larger-diameter stems than do deer, and will also destroy the growing points of stouter saplings by scratching themselves against stems [27]. The majority of oak population studies have focused on blue oak, followed by valley oak, and only a few studies exist on coast live oak [21]. Given the fact that coast live oak has tougher leaves and the leaves’ margins are armed with spiny margins, it is possible that mammalian herbivory on this species might be attenuated and therefore grazing by cattle and ungulates might not be such a critical factor in sapling survival. At any rate, research focusing on the seedling and sapling stages of coast live oak is necessary to assess the role of mammalian damage to understand the dynamics of oak recruitment limitation in this understudied specie.

Most studies of California oak woodlands and European Mediterranean oak stands are limited to a small spatial scale [21], [28]. These studies are appropriate for understanding ecological mechanisms that operate over short time intervals in limited space, such as factors impacting germination, or the effect of microsite on early survival and growth [18]. However, small spatial scale studies cannot adequately describe recruitment patterns at the stand level [29].

To address these gaps in knowledge, the present study focuses on oak regeneration at a regional scale in Northern California rangelands. We examined *Q. agrifolia* regeneration as a function of the presence or absence of cattle in different zones within a geographic area of ca 10,000 km^2^. These zones had different livestock management (cattle and wildlife presence vs. only wildlife). We focused on seedlings and saplings in coast live oak rangelands. Based on what is known for other species, we hypothesized that cattle management inhibits coast live oak regeneration via grazing. Specifically, we predicted that: 1) adult tree and young plant (seedlings and saplings) density would be lower in the presence of cattle; 2) the probability and intensity of herbivory would be greater in the presence of cattle and would vary with plant size; and 3) young oak plants sizes (as a proxy for young oak age) would be smaller in populations were cattle is present.

## Materials and Methods

### Ethics Statement

All the work was conducted in accordance with national and international guidelines, and conforms to the legal requirements of the California government (USA). Stanford University, Open Space Preserve, East Bay Regional Park District, Hastings Natural History Reservation and Vince Voegeli (private owner) granted permission to conduct the study in all selected ranches. Field studies did not involve endangered or protected species. Animals were only observed in the field, and were not captured or harmed in any way. Data are available.

### Study area

**Figure 1:**
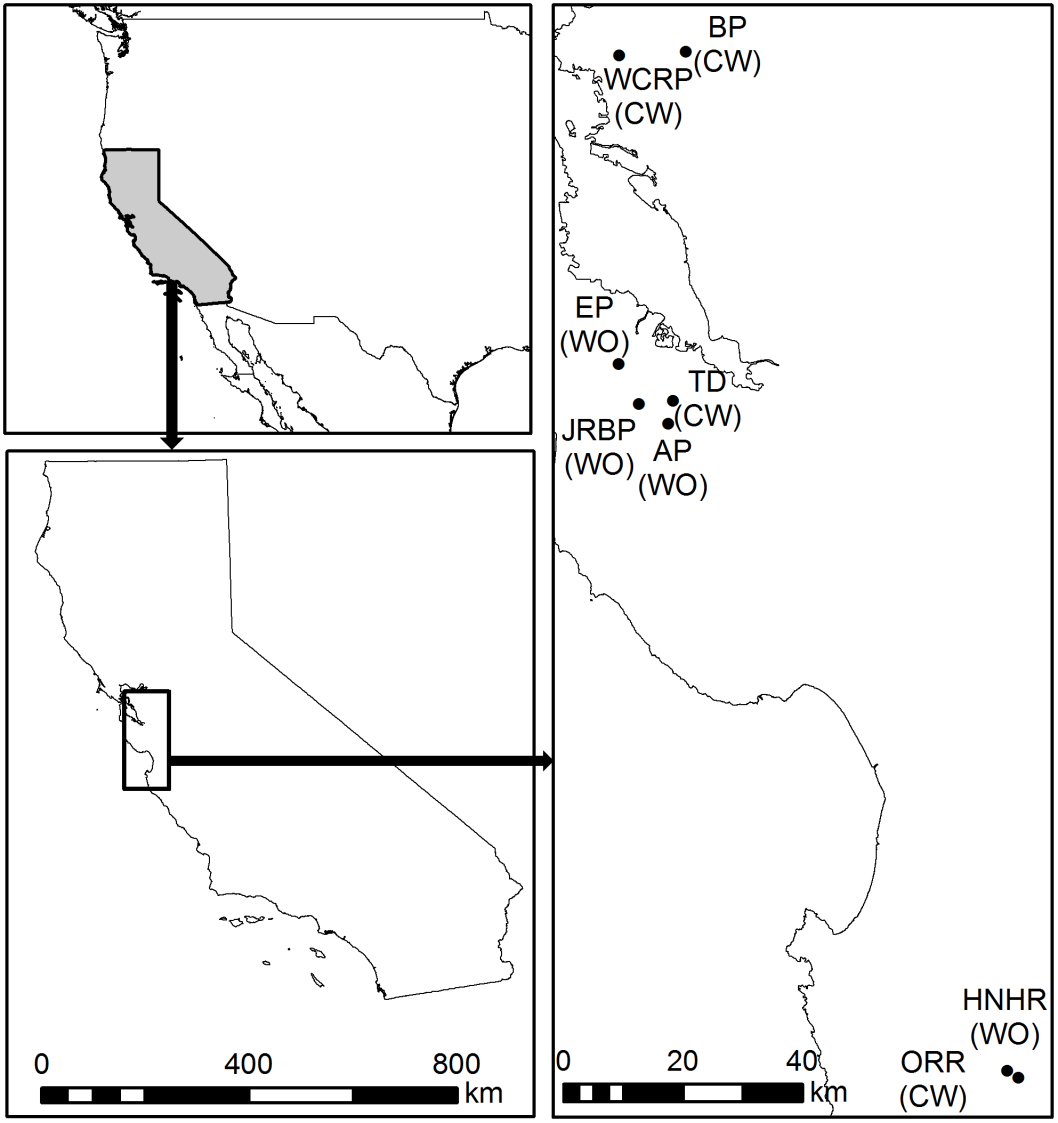
Location of the sampling ranches of coast live oak populations in Northern California, USA. JRBP, Jasper Ridge Biological Preserve; EP, Edgewood Preserve; AP, Enid Pearson-Arrastradero Preserve; HNHR, Hastings Natural History Reservation; ORR, Oak Ridge Ranch; TD, The Dish at Stanford University; BP, Briones Regional Park; WCRP, Wildcat Canyon Regional Park. The management condition of the study sites is indicated in parenthesis: CW, cattle and wildlife; WO, wildlife only.

The study was conducted across eight ranches located in Northern California, in 2013 (Figure 1). In all ranches, predominant vertebrate herbivores (mainly black-tailed deer (*Odocoileus hemionus* Rafinesque)) were present. Livestock has been maintained for at least some period in all selected ranches. Four of them have had cattle for more than 100 years; three of them currently support cattle year round, and one supports cattle from November to May. The rest have not supported cattle in the last 40 years (Table 1). The study area has a Mediterranean climate typical of California's coastal area, with dry, hot summers in which temperatures can reach 37.7°C. However, summer temperatures are usually tempered by coastal advection fogs providing significant moisture to plant communities. The 30-year average annual rainfall of the study area is 625.3 mm, concentrated in winter months, though inter-annual variation is considerable. Soils at the study sites are of metamorphic and sedimentary origins, maintaining complex substrate distributions that support a mosaic of plant community types, but open grasslands and oak woodlands of varying canopy coverage dominate the area’s landscapes. Given the latitudinal position of the study area, aspect also affects the microclimatic conditions and therefore vegetation structure and composition. Within each ranch, we selected sites defined as open oak woodland dominated by cost live oak (*Quercus agrifolia* Née). Study sites ranged in size from 160 to 243 ha (Table 1). Sites with homogeneous characteristics (largely flat and open) were selected for study.

**Table 1:** Location and main features of the ranches where coast live oak populations were sampled.

Ranch	County	Latitude (N)	Longitude (W)	Study area (ha)	Livestock
JPBR	San Mateo	37°24′	122°13′	175	No (40)
EP	San Mateo	37°28′	122°17′	188	No (46)
AP	Santa Clara	37°22′	122°11′	237	No (43)
HNHR	Monterey	37°23′	122°33′	243	No (76)
ORR	Monterey	37°23′	122°33′	160	Yes
TD	Santa Clara	37°23′	122°10′	228	Yes
BRP	Contra Costa	37°56′	122°08′	201	Yes
WCRP	Contra Costa	37°56′	122°17′	189	Yes

Values in parentheses indicate the number of years livestock have been absent, where applicable. JRBP, Jasper Ridge Biological Preserve; EP, Edgewood Preserve; AP, Enid Pearson-Arrastradero Preserve; HNHR, Hastings Natural History Reservation; ORR, Oak Ridge Ranch; TD, The Dish at Stanford University; BP, Briones Regional Park; WCRP, Wildcat Canyon Regional Park.

### Sample design and data collection

Nine 4 m×50 m belt transects were established within each ranch (total n = 72) and located in open coast live oak zones with an average size of 200 ha (Table 1). The belt transects were located in flat areas dominated by open annual grasslands (coverage = 70%±1.0776), oak woodlands of low canopy coverage (coverage = 20%, mean diameter = 56.7 cm, density = 102.8 trees.ha^−1^), and some shrub cover (<1% ±0.498). For each transect we recorded all adult and young plants, and measured all young plants. Seedlings and saplings were operationally defined as follows: plants with basal diameter less than 1 cm were considered seedlings; plants with basal diameter greater than 1 cm were considered saplings, which in turn were grouped into two height categories: below 50 cm (hereafter small saplings) and between 50 and 130 cm (hereafter large saplings). Vertebrate herbivory was assessed for all young plants. Damage was categorized according to its intensity. Damage categories were: 0 for plants with no apparent browsing evidence, 1 for plants with low browsing evidence (1–10% of the browsable biomass damaged), 2 for plants with moderate browsing intensity (11–40% of the browsable biomass damaged), 3 for plants with considerable browsing intensity (41–70% of the browsable biomass damaged), and 4 for plants with high browsing intensity (>70% of the browsable biomass damaged). These scorings were calibrated among researchers for consistency by initially practicing together and arriving to consensus scoring before scoring plants independently. The surveys were conducted during April, 2013.

### Variables analyzed and statistical analysis

Data processing and statistics were performed using R 2.13.2 (R Development Core Team, 2012) with the modules “lme4” [30], “car” [31] and “nnet” [32].

In order to analyze oak density as a function of rangeland management, we developed two generalized linear mixed models (GLMM, using the model approach in [33]): (1) using mature trees per plot as the response variable, and (2) using number of young plants (seedlings plus saplings) as the response variable. We also analyzed seedlings and saplings as response variables independently, but results did not differ from those including both. Analyses included ranch nested within treatment as a random effect, and treatment as fixed effect. Treatment has two levels: presence of cattle and wildlife, and presence of only wildlife. Latitude and longitude (for each transect) were included as predictor covariates in order to consider and control for other potentially important factors -related to the spatial location- affecting oak density. The interactions between all terms were included as well. We selected the best model based on Akaike's Information Criterion (AICc [34]). Both number of adult trees and number of young plants were fitted to Poisson error distributions with a log link function.

In addition to plant density, occurrence of herbivory was analyzed as a response variable by means of a GLMMs. Beyond herbivory occurrence, intensity of herbivory was analyzed using herbivory categories (described in part 2.2) by means of a multinomial log-linear mixed model [32]). The analyses considered transect nested within ranch nested within treatment as random effects. Models included treatment (presence or absence of cattle) and diameter of plant as fixed effects. Latitude, longitude, and the interactions between all terms were included as predictor covariates. Occurrence of herbivory was fitted to a binomial error distribution with a logit link, and the intensity of herbivory was fitted to a multinomial error distribution with a cumulative logit link. One more GLMM was developed using diameter of young plants as the response variable. Analyses included the same random effects as those previous models that have the occurrence and intensity of hervibory as response variables. Models included treatment and occurrence of herbivory as fixed effects. Plant diameter was fitted to a gamma distribution with logit link. AICc was used for model selection [34] in all cases.

## Results

### Density of oak plants depending on the presence of cattle

**Figure 2:**
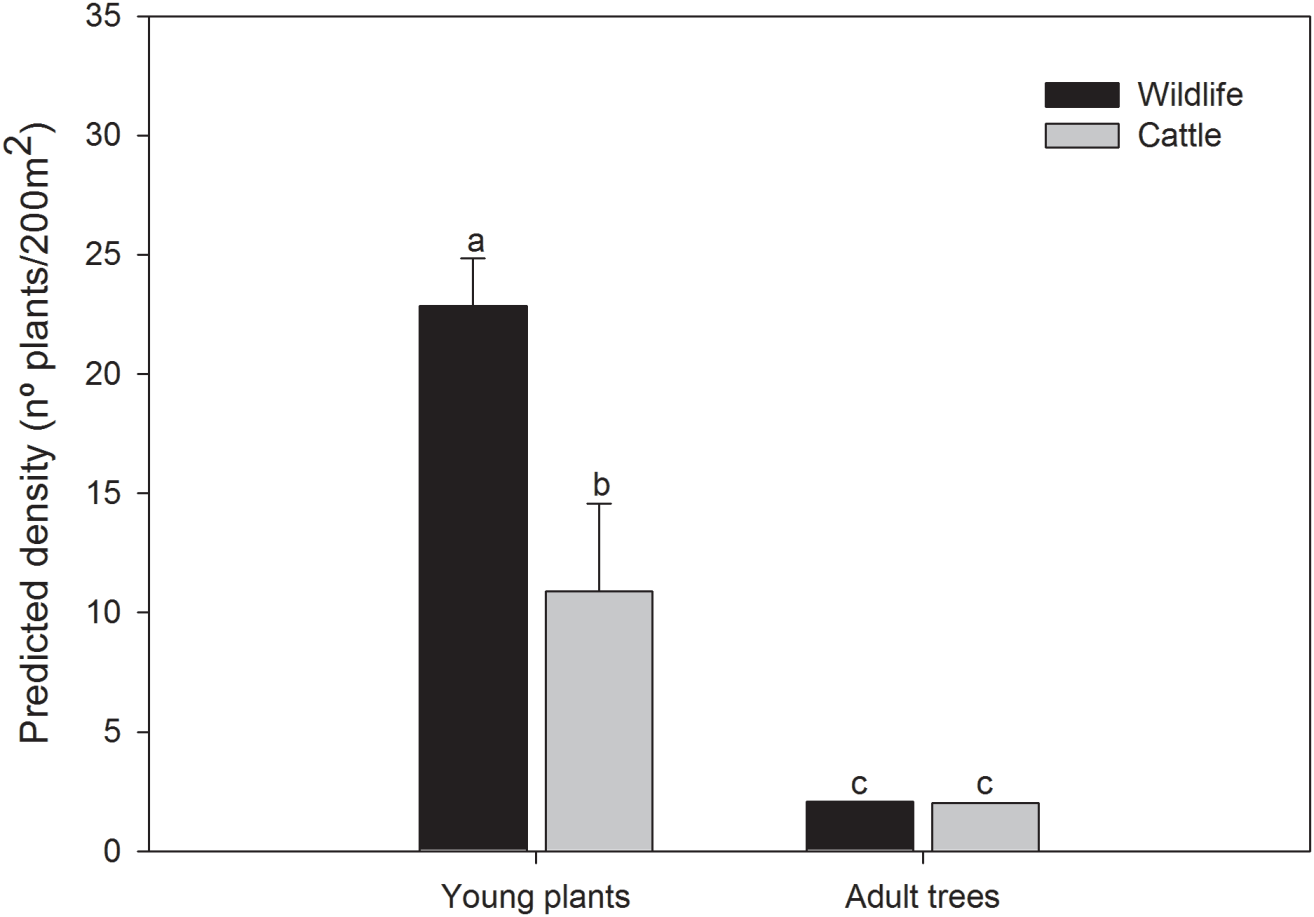
Density of different oak class sizes. Predicted density of different class sizes found at our study depending on the presence or absence of cattle (N = 72 sampling plots). Error lines are 95% confidence intervals. Note that in the case of adult trees the confidence intervals for predicted values are not visible because of their small size. Young plants: seedlings (plants with basal diameter less than 1 cm) and saplings (plants with basal diameter>1 cm and height<130 cm); Adult trees: plants with basal diameter>7.5 and height>130 cm.

The density of adult trees did not differ depending on the presence or absence of cattle, location of the plot (latitude and longitude), or management type of the ranch (Table 2). However, we found that the density of young plants, both seedlings and saplings, did differ depending on the presence or absence of cattle (Table 2). The results of independent models for seedlings and saplings were identical to the results of the models taking all young plants together, thus we only show the results using all young plants together for simplicity. In areas where cattle were present in addition to wildlife, the density of young plants was less than half (around 10 young plants per 200 m^2^) than in areas in which only wildlife was present (more than 20 young plants per 200 m^2^) (Figure 2). Additionally, we found significant effects of plot location on the density of young plants (Table 2).

**Table 2:** Summary of the Generalized Linear Mixed Models fitted for oak plant density (including adult trees (A) and young plants (B) – seedlings and saplings) as the response variable.

Response variable	Variables	Variance	*SD*	*Coeff.*	*SE*	*z-*value	*P*
Density of adult trees (A)	*Random effects*						
	Ranch	0.000	0.000				
	*Fixed effects*						
	Treatment (Wildlife)			0.026	0.166	0.161	0.872
Density of young plants (B)	*Random effects*						
	Ranch	0.151	0.389				
	*Fixed effects*						
	Treatment (Wildlife)			2.332	0.381	6.108	<0.001
	Latitude			3.539	0.761	4.657	<0.001
	Longitude			−6.017	1.429	−4.244	<0.001

### Probability and intensity of herbivory depending on the presence of cattle

**Figure 3:**
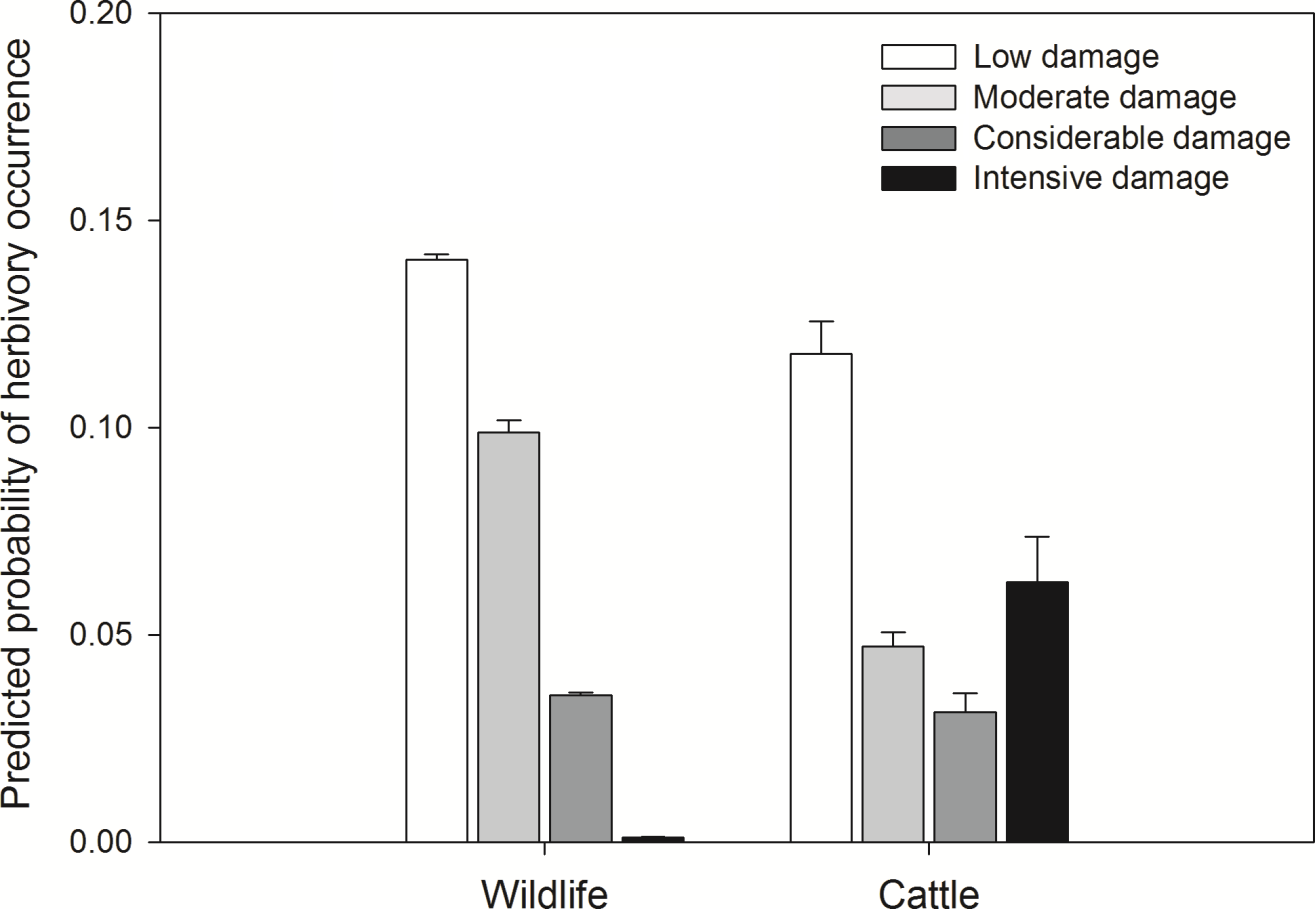
Predicted probability of herbivory occurrence. Predicted probability of plant being grazed across different damage categories depending on the presence or absence of cattle (N = 1,201 plants). Error lines are 95% confidence intervals. Low damage = 1–10% of browsable biomass was damaged; Moderated damage = 11–40% of browsable biomass was damaged; Considerable damage = 41–70% of browsable biomass was damaged; Intensive damage = >70% of browsable biomass was damaged.

The probability of the occurrence and intensity of herbivory depended strongly on plant spatial location, plant diameter, and the presence or absence of cattle (Tables 3, 4). Plants with larger diameters showed a higher probability of been grazed (Table 3). When cattle were present the probability of presenting herbivory damage was higher (Table 3) and damage scores were also higher (>70% of the browsable biomass was damaged) than when only wildlife was present (Figure 3). The effect of diameter on the probability of occurrence of herbivory did not depend on the presence of cattle, suggesting that both wildlife and cattle show similar preferences for plant sizes.

**Table 3:** Summary of the Generalized Linear Mixed Models fitted for occurrence of herbivory as the response variable.

Response variable	Variables	Variance	*SD*	*Coeff.*	*SE*	*z-*value	*P*
Probability of herbivory’s occurrence	*Random effects*						
	Transect	2.250	1.500				
	Ranch	0.526	0.725				
	*Fixed effects*						
	Treat. (Wildlife)			−3.714	1.069	−3.473	<0.001
	Diameter			0.028	0.010	−2.708	0.006
	Latitude			−7.880	2.197	−3.587	<0.001
	Longitude			0.136	4.182	3.261	0.001

Treat: Treatment.

**Table 4:** Summary of the multinomial log-linear mixed models (Likelihood-ratio *χ2* test) fitted for intensity of herbivory as the response variable.

Response variable	Variables	*d.f*	*LR χ2*	*P*
Intensity of herbivory	*Random effects*			
	Transect	4	1.336	0.855
	Ranch	4	1.336	0.855
	*Fixed effects*			
	Treatment	4	128.561	<0.001
	Diameter	4	10.057	0.039
	Latitude	4	86.852	<0.001
	Longitude	4	77.787	<0.001

### Diameter of oak plants depending on the presence of cattle

**Figure 4:**
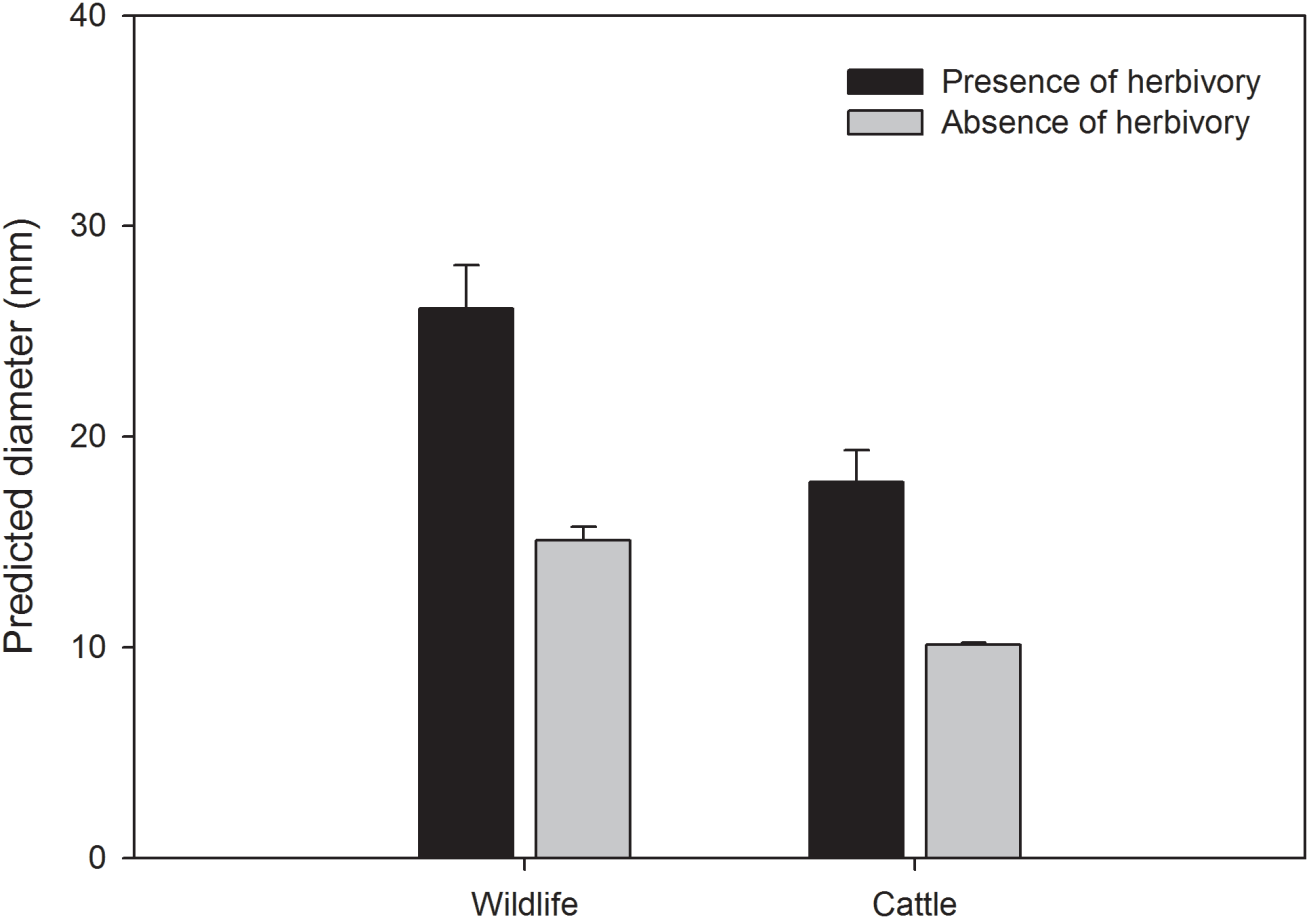
Plant size. Predicted plant diameter depending on the presence or absence of herbivory and of cattle (N = 1,201 plants). Error lines are 95% confidence intervals.

Plant diameter depended on the ranchs management type. Seedlings and saplings were smaller in ranches where both cattle and wildlife were present than in ranches where only wildlife was present (Figure 4). Larger plants were more grazed (Figure 4) and the size difference between grazed and ungrazed plants was stronger when only wildlife was present.

## Discussion

Lack of adequate regeneration is a major problem faced by woodland oak species in California, especially in cattle rangelands [21], [35]–[37]. Through this study we compared the densities of adult and young plants, observing that presence of cattle produced a notably change in young oak populations. Besides oak recruitment limitation associated with rangeland management, herbivory pressure on plants, depending on the presence of cattle, seem to vary as well. We focused specifically on coast live oak (*Q. agrifolia*), which shows recruitment levels insufficient for maintaining oak stands in certain areas [13], [23]. Coast live oak populations show low recruitment especially in open areas dominated by mostly herbs and a few shrubs [23], [38]. In Mediterranean climates, herbaceous vegetation gets completely consumed or dried during summer [39], making ungulates to switch food sources. Hence, late season use of browse as a food resource by ungulates and cattle increases, and subsequently affects oak regeneration [40], [41]. An increase of drought due to climate change not only would prompt a decrease of hydric resources and consequently would generate difficulties in the normal growth of the vegetation [42]; but also would exacerbate grazing pressure on browse, especially over young plants [41]. Given the potentially high herbivory levels at early ontogenetic stages of plants, herbivory of coast live oak seems to be an important factor driving regeneration rate, although other factors have been found to also be important e.g., substrate type, soil moisture levels, shade, fire regime, and drought frequency [43], [44]. Moreover, heavy grazing over many years can indirectly affect oak recruitment by increasing soil compaction and reducing organic matter [45], thereby making it more difficult for oak roots to penetrate downward to obtain moisture [45]. While cattle’s grazing has been blamed for poor oak recruitment in rangelands, removal of livestock has often failed to alleviate oak recruitment problems even after many years without grazing (e.g., [46], [47]). In our study, we observed a reduction in the density of coast live oak seedlings and saplings, but not adults - (more than 100 years old [48], [49]) - in areas grazed by cattle relative to seedling and sapling density in abandoned areas (at least 40 years without livestock), corroborating previous similar findings [50] in Israel. The presence of cattle for a long period negatively affected oak regeneration, especially in pastures with a high stocking rate. The mobility of wildlife reduced such constant grazing pressure, allowing more oak recruitment than in permanently stocked rangelands [51].

In other agroforestry systems, some measures have been proposed to promote the maintenance of the tree layer and to alleviate regeneration problems such as transhumance (traditional livestock movements through grazing zones and seasons, at the landscape level), or temporary absence of livestock in Spanish (Mediterranean) agroforestry systems (“dehesas”) [52], [53]; or adoption of non-continuous grazing management schemes in Australian rangelands [54]. These studies have been used to justify the increase of livestock mobility in California rangelands as a way to allow increased oak regeneration.

In the present study, differences in browsing depending on ranchs management type (wildlife vs. cattle and wildlife) affected plant density in populations of young coast live oak plants. Moreover, plants showed a larger diameter in areas were cattle were not present. These differences in plant diameter indicate that in areas where cattle are present the opportunity of the plants to pass on to the next ontogenetic stage would be reduced. We found intensive damage (>70% of browsable biomass damaged) in cattle areas. The smaller diameter of the young plants in cattle grazed areas compared to areas without cattle (Figure 4) could result from a reduction in the net growth rate of oak juveniles due to the intensity of damage caused by cattle, which could also reduce the transitioning from seedlings to saplings. Our findings suggest that the likelihood of young coast live oak individuals reaching the subsequent size classes is significantly reduced in cattle ranches, especially during transition from small saplings to the next phase of saplings. In contrast, in the Spanish dehesas, unsuccessful holm oak (*Quecus ilex* L.) recruitment seems to be limited not by transition between sapling stages but by failures of seedling emergence and establishment [55]. Unsuccessful transition from the seedling to sapling phase can be a critical determinant of the lack of oak regeneration [51]. In both types of management (wildlife only vs. cattle and wildlife), grazers showed similar preferences for plant sizes. However, the grazing pressure over plants seem to vary depending on the community of animals grazing on them, with cattle frequently chewing into larger diameters for longer grazing periods thus causing more shoot damage than do deer [27].

Field studies on oak recruitment that are conducted at larger spatial (the present study) or temporal scales can contribute information about the potential ranges of age-specific survivorship for a given set of environmental conditions, and about the relative importance of limiting factors for different life stages [18]. We have found important effects of the spatial locations of the plots (latitude and longitude) on the density of young plants, which might indicate that the effect that animals have on the regeneration of this species is also related to their spatial location. Woodlands of *Q. agrifolia* in cooler, moister climates in northern California contained proportionally more saplings than woodlands in southern California [56]. Increasing the number of study sites across a greater spatial range within Northern California would improve our ability to accurately model oak population dynamics, to understand controls on oak demography, and to predict outcomes of restoration and other management efforts [18].

Decades of research have produced no definitive conclusion about the existence of the California oak ‘regeneration problem’ [21]. The underlying causes of perceived recruitment failure are unclear and could include drivers at broad scales including such as climate change, habitat fragmentation, altered herbivore populations, changes in fire regimes, exotic plant and animal invasions, livestock grazing, and soil conditions altered by past land uses [21]. In our study we have not included microhabitat effect, or competition with annual grasses, all of which may be important determinants of the regeneration stage of coast live oak at a short spatial scale. Further research on the conditions that would ensure tree regeneration in Californian rangelands is needed in order to determine thresholds of grazing density and timing. Moreover, there remains a lack of information about the factors that govern the transitions from seedlings to saplings [57], [58], and from saplings to adults [59], [60], because the majority of oak regeneration studies [21] have focused on the earliest ontogenetic stages only (e.g., [24], [61], [62]). Therefore, further research about the factors determining the transition probability to reach subsequent ontogenetic stages will be necessary to understand better the regeneration problem.

Permanent exclusion of cattle from rangelands may not be an optimal management solution, as light to moderate cattle grazing enhances species richness among herbaceous species, prevents fire hazards by reducing encroachment of dwarf shrubs [50], reduces competition with annual grasses [61]–[63], and reduces herbivory by small animals attracted to high herbaceous cover [64], [65]. Cessation of grazing would also have negative economic implications for ranchers [50], [54], [66]. Movement of cattle around the ranch might help to more evenly distribute grazing pressure. Compared with deer, browsing pressure from fenced cattle is generally more intense, more consistent, and consequently more damaging to oak regeneration [27]. Transhumance is a millenary practice that has been proven to provide a wide range of ecosystem services and preserve the cultural landscapes of Spanish dehesas by allowing sustainable regeneration of the tree structure [51]. Changing the position of feeding points over time and distributing several water points throughout the ranch would encourage the movement of cattle. Grazing cessation during summer and autumn [51], reduction of stocking rate, deferment of the commencement of grazing, and fencing young seedlings [50] can be applied to improve the regeneration of scattered trees. In the face of inadequate recruitment, management practices must be modified to allow for the establishment of new trees.

The scattered trees of open woodlands that characterize these landscapes are widely recognized as keystone organisms due to the multiple ecological processes that depend upon their presence [54], [66]. We found that the density of seedlings and saplings in California’s rangelands was 50% lower in grazed sites compared to ungrazed sites. Under significant grazing pressure by cattle, young coast live oak plants may rarely survive beyond the seedling and sapling size classes. Given the importance of Californian rangelands, it is increasingly critical to employ science-based measures to better manage and ensure the continuity of these landscapes.
